# Heterogeneity in Genetic Diversity among Non-Coding Loci Fails to Fit Neutral Coalescent Models of Population History

**DOI:** 10.1371/journal.pone.0031972

**Published:** 2012-02-22

**Authors:** Jeffrey L. Peters, Trina E. Roberts, Kevin Winker, Kevin G. McCracken

**Affiliations:** 1 Department of Biological Sciences, Wright State University, Dayton, Ohio, United States of America; 2 University of Iowa Museum of Natural History, Iowa City, Iowa, United States of America; 3 University of Alaska Museum, University of Alaska Fairbanks, Fairbanks, Alaska, United States of America; 4 Institute of Arctic Biology, University of Alaska Fairbanks, Fairbanks, Alaska, United States of America; Brigham Young University, United States of America

## Abstract

Inferring aspects of the population histories of species using coalescent analyses of non-coding nuclear DNA has grown in popularity. These inferences, such as divergence, gene flow, and changes in population size, assume that genetic data reflect simple population histories and neutral evolutionary processes. However, violating model assumptions can result in a poor fit between empirical data and the models. We sampled 22 nuclear intron sequences from at least 19 different chromosomes (a genomic transect) to test for deviations from selective neutrality in the gadwall (*Anas strepera*), a Holarctic duck. Nucleotide diversity among these loci varied by nearly two orders of magnitude (from 0.0004 to 0.029), and this heterogeneity could not be explained by differences in substitution rates alone. Using two different coalescent methods to infer models of population history and then simulating neutral genetic diversity under these models, we found that the observed among-locus heterogeneity in nucleotide diversity was significantly higher than expected for these simple models. Defining more complex models of population history demonstrated that a pre-divergence bottleneck was also unlikely to explain this heterogeneity. However, both selection and interspecific hybridization could account for the heterogeneity observed among loci. Regardless of the cause of the deviation, our results illustrate that violating key assumptions of coalescent models can mislead inferences of population history.

## Introduction

DNA polymorphisms provide an invaluable means to study the influence of historical processes that shape genetic diversity, such as divergence times, gene flow, and fluctuations in population sizes. To increase the statistical rigor by which these processes are inferred, the field of phylogeography has advanced in two directions. First, coalescent theory [1], [2] is now routinely applied in phylogeographic studies. Coalescent methods incorporate the stochastic variance of genetic processes by estimating parameters from many genealogies consistent with the data, and thus provide a framework for testing competing hypotheses while accounting for uncertainty (i.e., confidence intervals) in parameter estimates [3], [4]. Second, estimating parameters from multiple independent loci has become common [5]–[12]. A multilocus approach has been motivated by two fundamental problems with single-locus studies: the stochasticity of mutation and genetic drift creates variable signatures in DNA even when different loci experienced identical population histories [3], [13], [14], and single-locus studies do not adequately address the possibility that selection, not population history, has generated patterns in DNA [15]–[17]. Because mutation, drift, and selection operate independently on unlinked loci, applying coalescent methods to multiple loci can strengthen inferences of population history.

Although coalescent methods and multilocus approaches have advanced the field substantially, there are still a number of challenges to be addressed. Among them is how well the genetic data fit the coalescent models used to infer population histories [18], [19]. Actual population histories are usually, if not always, more complex than the available models, and they can violate any number of simplifying assumptions. Common assumptions in analytical programs using coalescence include constant or exponentially-changing effective population sizes (*N_e_*), constant migration rates over time, panmictic populations that do not exchange genes with other populations, simple models of molecular evolution, and selective neutrality [20]–[22]. Simulation studies demonstrate that violating these assumptions can sometimes bias parameter estimates [23]–[25]. Therefore, understanding how well empirical data fit these models is necessary to obtain robust inferences of population history and to understand the influences of selection and other processes. Although coalescent methods can be incredibly flexible, and additional relevant parameters can be added [26], doing so increases computational demands and requires additional data (e.g., more loci) to obtain sufficient signal in the DNA.

Empirical studies have revealed that heterogeneity in the patterns of genetic diversity can be substantially higher than expected under simple, neutral models of population history, which is attributed to more complex demographic histories or selection [27]–[32]. Distinguishing between these scenarios is difficult, because patterns generated by different forms of selection can mimic the patterns generated by various population histories [15], [33], [34]. A key to disentangling the effects of population history and selection is that population history affects loci throughout the genome in a similar fashion, whereas selection only affects the locus (or loci) under selection and those that are closely linked. Thus, population history generates similar patterns of DNA polymorphisms throughout the genome, whereas selection has a local effect causing idiosyncratic patterns among loci [34]–[36]. However, some forms of demographic history, such as bottlenecks, can cause heterogeneous patterns among loci that are difficult to distinguish from the effects of selection [27], [28], [30], [31], [37]. Furthermore, if selection is pervasive throughout the genome, it might have a strong net effect on our ability to infer population histories.

In this study, we tested the fit of non-coding DNA sequence data sampled from a genomic transect (∼1 locus per chromosome; 22 loci) in a species of duck, the gadwall (*Anas strepera*), to two popular coalescent models: the two-island model from the program lamarc [22] and the isolation-migration model from the program im [20]. We then used coalescent simulations to test three hypotheses that might explain the poor fit between empirical data and the models, including a pre-divergence bottleneck, interspecific hybridization, and selection. Because there are an infinite number of complexities that could contribute to empirical data deviating from the models, these hypotheses are not intended to be exhaustive. Rather, we focus on these three hypotheses because we suspect *a priori* that these factors might have had a prominent influence on measures of genetic diversity.

## Materials and Methods

### Study Taxon

**Figure 1:**
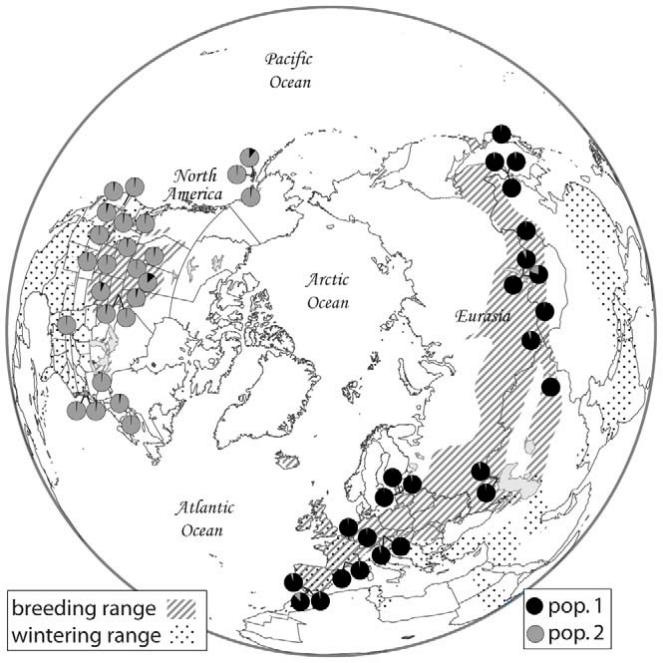
Sampling locations and population assignment probabilities. Assignment probabilities are based on genotypes from 22 non-coding loci for 50 gadwalls. Note that all 25 individuals sampled from the OW were assigned to population 1, and all 25 individuals sampled from the NW were assigned to population 2 with high assignment probabilities.

The gadwall has a Holarctic distribution extending across Eurasia and North America (Fig. 1). Range disjunctions created by the Atlantic and Pacific oceans subdivide the gadwall into two allopatric populations that are genetically differentiated [10], [38]. An Old World (OW) population occurs from Spain to Japan, and a New World (NW) population occurs from Alaska to the east coast of North America. Genetic evidence suggests that population structure within continents is limited to a few peripheral populations that differ from the remaining populations in mitochondrial DNA (mtDNA) haplotype frequencies [10], [38], but that nuclear DNA (nuDNA) is consistent with a single panmictic population within each continent [10]. These data also suggest that gadwalls colonized North America from Eurasia during the Pleistocene, and that these two populations are connected by moderate levels of gene flow [10].

### DNA Sequencing

We sampled 25 OW and 25 NW gadwalls from widely distributed locations across North America, Europe, and Asia (Fig. 1; individuals were subsampled from the dataset of Peters et al. [10]). We also sampled seven species as outgroups to examine relative substitution rates among loci; these seven species were the snow goose (*Anser caerulescens*), ruddy duck (*Oxyura jamaicensis*), musk duck (*Biziura lobata*), pink-eared duck (*Malacorhynchus membranaceus*), black-bellied whistling duck (*Dendrocygna autumnalis*), magpie goose (*Anseranas semipalmata*), and southern screamer (*Chauna torquata*). These species represent the major clades of waterfowl (Order Anseriformes) that are all deeply divergent from each other [39]. They are close enough genetically for reasonable sequence alignment, but distant enough to reduce the effects of differential sorting of ancestral polymorphisms on estimates of long-term substitution rates (see below for additional details).

For each individual, we obtained nuclear DNA sequences for 22 non-coding loci, including 21 introns and 1 microsatellite locus, covering more than 7 kbp of sequence data and mapping to at least 19 different chromosomes in the chicken (*Gallus gallus*) genome [40], [41] (Table 1). Five of these loci had been published previously [10], seven loci were chosen because primers had been developed for other studies of ducks [42]–[44] (M. Sorenson unpubl. data), and ten loci were found by searching GenBank for intron or mRNA sequences isolated from ducks. Our primary requirement for selecting a new locus was that it be linked to a different chromosome in chickens, but we also targeted shorter introns when available sequence from ducks was limited to mRNA (intron length and location was estimated from the chicken genome). We chose all loci blindly with respect to levels of polymorphism. When designing primers, we used both duck and chicken sequences, and therefore our primers will likely be useful for studies of other birds.

**Table 1:** Characteristics of the 22 non-coding loci sequenced in gadwalls.

Locus	Abbreviation^1^	Location^2^	Intron #	Length (bp)	*π* 3	*Φ_st_* 3	Tajima's *D* 3
Chromo-helicase-DNA binding protein gene 1	CHD1Z	Z/Z	19	270	0.0015	0.038*	−0.67
Lactate dehydrogenase 1	LDHB	1/1A	3	460–461	0.0008	0.143***	−0.96
T-cell surface glycoprotein CD4 (surface antigen)	CD4	1/1	5	345	0.0010	−0.002	−2.14**
S-acyl fatty acid synthase thioesterase	FAST	2/2	2	319–322	0.0027	0.026	−1.13
Ornithine decarboxylase	ODC1	3/3	5	275–295	0.0143	0.010	−0.66
Fibrinogen beta chain	FGB	4/4	7	433	0.0076	0.032*	−0.61
Serum amyloid A	SAA	5/5	2	347–351	0.0195	0.053**	−0.22
Annexin A11	ANXA11	6/6	5	405	0.0061	0.132***	−0.41
Myostatin	MSTN	7/7	2	291	0.0175	0.026*	−0.20
Sterol O-acyltransferase 1	SOAT1	8/?	12	352	0.0071	−0.014	0.72
Nucleolin	NCL	9/9	12	329–341	0.0260	0.068**	0.69
Lecithin-cholesterol acyltransferase	LCAT	?/11	2	308–339	0.0215	0.036**	0.66
Preproghrelin	GHRL	12/?	3	348–351	0.0220	0.191***	0.10
Glutamate receptor, ionotropic, N-methyl D aspartate I	GRIN1	17/17	11	290	0.0004	0.014	−1.28
Sex determining region Y-box 9	SOX9	18/18	2	352–381	0.0295	0.045***	−0.04
Carboxypeptidase D	CPD	19/19	9	304–328	0.0293	0.092***	1.11
Phosphenolpyruvate carboxykinase	PCK1	20/20	9	324–325	0.0036	0.001	0.43
Alpha enolase 1	ENO1	21/21	8	164–175	0.0062	0.089***	−1.03*
Alpha-B crystallin	CRYAB	24/24	1	294	0.0015	0.147**	−0.14
Growth hormone 1	GH1	27/1?	3	363	0.0018	0.005	−0.33
Splicing factor 3A subunit 2	Sf3A2	28/?	8	305	0.0004	0.007	−1.43*
Tetranucleotide microsatellite repeat A27E1	A27E1	?/?	NA	171–183	0.0027	0.292***	−0.67*

1 Locus abbreviations follow standards put forth by the Chicken Gene Nomenclature Committee [41].

2 Chromosomal location within the chicken genome and the zebrafinch genome, respectively. ? = unknown.

3 Summary statistics exclude regions containing gaps;

*p<0.05,

**p<0.01,

***p<0.001.

The 17 new loci were amplified using standard PCR protocols with an annealing temperature of 58°C and 45 cycles (primer sequences are available in Supporting Information, Table S1). Sequencing was performed using the Big Dye v.3.1 sequencing kit (Applied Biosystems) and direct sequencing was done using an ABI 3100 automated sequencer (Applied Biosystems). Sequences were edited using Sequencher software (Gene Codes, Ann Arbor, MI); all sequences have been deposited in GenBank (Accession numbers JQ180538–1538, JQ255480–5607). When available, outgroup sequences published in GenBank were used (Supporting Information, Table S2) [39], [42], [45]–[48]. Loci were initially aligned in Sequencher, but loci containing indels that could not be unambiguously aligned (CPD, LCAT, NCL, SAA, and SOX9) were aligned using ClustalW in the program MEGA 5.0 [49]. Outgroup sequences were also aligned in MEGA 5.0.

We resolved the gametic phase of alleles using three methods. First, sequences containing indels were resolved by comparing the ambiguous 3′ end of the forward strand with the unambiguous 5′ end of the reverse strand, and vice versa, to determine the length and composition of the gap region. Because indels result in shifted peaks in the chromatograms, it was possible to determine which polymorphisms throughout the sequence were linked to the gap [50], thus resolving the gametic phases. Seventy-two sequences were heterozygous for multiple indels, and we designed allele-specific primers that targeted either a single nucleotide polymorphism or the indel itself to preferentially amplify and sequence each allele independently to resolve those alleles. Second, we used the program phase to reconstruct the most likely gametic phase of each heterozygous sequence [51]. phase input files were created using the program seqphase [52]. Third, when the probability of reconstructed alleles was less than 0.95, we used allele-specific primers to amplify and sequence one of the two alleles independently and then subtracted this allele from the heterozygous sequence to resolve the gametic phase of the other allele [53]. We then repeated PHASE analyses, with the newly resolved alleles defined as known alleles to verify that all reconstruction probabilities were ≥0.95. In total, 289 of the 850 new sequences (34%) were resolved using allele-specific priming. Fasta files containing the resolved alleles for each locus are archived in dryad (datadryad.org; doi:10.5061/dryad.nv5v1v59).

### Delineating Populations

We estimated the number of genetic populations (*K*) and assigned individuals to those populations using the MCMC Bayesian method in the program structure v.2.2.3 [54], which uses deviations from Hardy-Weinberg equilibrium and linkage disequilibrium to examine population structure. We numbered alleles for each locus from 1 to *n*, where *n* is the total number of different alleles for that locus. We used an admixture model with allelic frequencies assumed to be independent and estimated Pr(*X*|*K*) for *K* = 1 to 5 populations. We then calculated Δ*K* [55], which has been shown to be a better estimator of the true *K* compared to Pr(*X|K*). No *a priori* information about sampling localities was included in these analyses. Each analysis was run for a burn-in of 10,000 generations followed by 20,000 generations of sampling. We replicated each run five times and report values averaged across all runs.

### Summary Statistics

We calculated the following parameters from the empirical data: *π* (nucleotide diversity within the total gadwall population), *Φ_st_* (the percentage of nucleotide diversity explained by differences between OW and NW gadwalls), and Tajima's *D* (a measure of the relative abundance of low-frequency polymorphisms). These parameters were calculated in the program DnaSP v. 4.50.3 [56] and Arlequin v3.11 [57]. We inferred haplotype networks using the median-joining algorithm in the program Network v. 4.5.1.0 [58]. Gaps were excluded from all analyses.

### Heterogeneous Substitution Rates

We tested for heterogeneous substitution rates using one arbitrarily selected gadwall sequence (UAM 18797) and each of the seven outgroup sequences. By using multiple outgroups that are deeply diverged from *Anas* ducks, we were able to account for the stochastic variance of mutation and lineage sorting in our estimates of substitution rates. We estimated relative substitution rates (*μ_R_*) among loci using the multispecies coalescent method in *beast [59]. All 22 loci were included in the analysis, and *μ_R_* for each locus was scaled to the average rate among loci. We ran *beast for 100,000,000 generations, sampling parameters every 10,000 generations and discarding the first 1,000 samples as burn-in. Based on preliminary analyses, we used uniform priors on *μ_R_* that ranged from 0.1 to 5 times the average (these priors were wider than the bounds on the posterior distribution from the preliminary analysis, and were therefore assumed to be uninformative). We used a relaxed lognormal molecular clock to account for the possibility of unequal rates among branches [60]. Our *beast input file has been submitted to dryad (doi:10.5061/dryad.nv5v1v59).

### Inferring Population History

**Figure 2:**
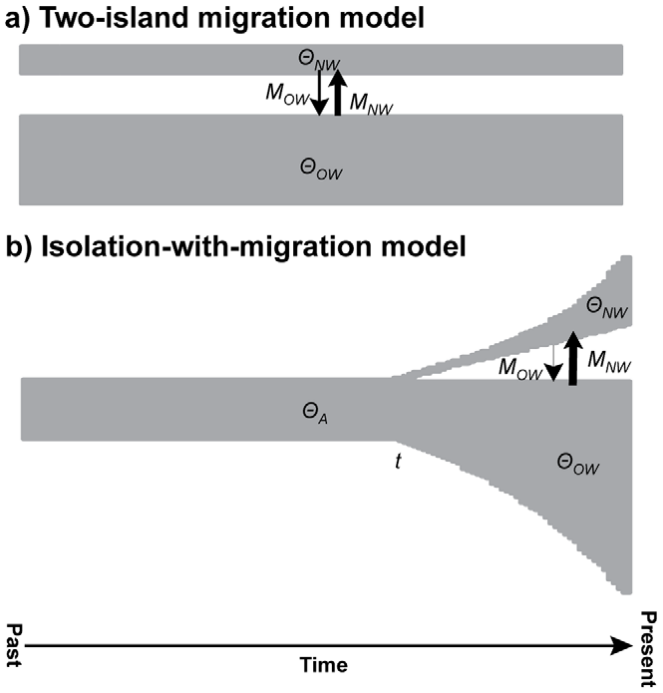
Basic population models. Illustrations of the two-island (a) and isolation-migration (b) models of population subdivision inferred in this study.

We used coalescent methods to infer the population history of gadwalls under two different models of population subdivision. The first model was a simple two-island migration model, whereby *N_e_* and migration rates were assumed to be constant over time and divergence times occurred infinitely in the past (Fig. 2a). We used the MCMC Bayesian method in the coalescent program lamarc v2.1.6 [22] to jointly estimate the parameters *Θ_i_* (where *Θ_i_* = 4*N_ei_μ*, and *N_ei_* is the effective size of population *i* and *μ* is the geometric mean of the per-site substitution rates among loci) and *M_i_* (where *M_i_* = *m_i_*/*μ*, and *m_i_* is the migration rate into population *i* from population *j*). Recombination was also incorporated into this analysis, and we used the Felsenstein 84 model of substitution (ti∶tv = 2.5; the average ratio among loci). Each locus was run independently for a burn-in of 2,000,000 generations followed by 20,000,000 generations sampling parameters every 1,000 generations (a total of 20,000 samples). Each run was replicated with a different random number seed to verify convergence among runs.

Parameters estimated in lamarc are scaled to the substitution rate per site (*μ*), and we adjusted these estimates using *μ_R_* for each locus calculated in the *beast analysis of the eight-taxon dataset (see above). To do this, we divided each estimate of the locus-specific *Θ* sampled from the posterior distribution by the locus-specific *μ_R_* randomly selected from the posterior distribution obtained from *beast. Likewise, we multiplied each value from the posterior distribution of *M* by a randomly selected value of *μ_R_*. Thus, our conversions incorporated uncertainty in *μ_R_*. Following the lamarc methods, we calculated joint estimates of *Θ* and *M* by multiplying the likelihoods among all loci after smoothing the distributions using a biweight kernel.

For the second model, we used the MCMC Bayesian genealogy sampler in the coalescent program im [20], [21] to infer a more complex isolation-migration model (Fig. 2b) that included joint estimates of *θ_i_* (where *θ_i_* = 4*N_ei_u*, and *u* is the geometric mean of the per-locus substitution rate), constant migration rates (where *M_i_* = *m_i_*/*u*), time since divergence (where *t* = *Tu*, and *T* is the number of generations that have passed since the populations split), ancestral *θ* (*θ*_A_) at the time of divergence, and population growth (*s* & 1−*s*; the proportion of the ancestral population contributing to each of the daughter populations). (Note that the different symbols are used to differentiate between parameters scaled to the substitution rate per site (*Θ*, *μ*) in lamarc versus those scaled to the rate per locus (*θ*, *u*) in im.) Because im does not accommodate recombination, we used the program imgc [61] to select an optimal fragment size consistent with no recombination by removing individuals and/or base pairs of data. We allowed a maximum of 5% of alleles (*n* = 5) to be removed from the analysis, which presumably allows for the removal of rare recombinants and PCR/editing errors without dramatically changing allele frequencies. We included all loci in a single im run with 40 chains and a burn-in of 1,000,000 generations. We then sampled parameters every 50 generations for at least 10,000,000 generations. The minimum ESS was 100 among parameters, and the analysis was replicated with a different random number seed to verify convergence.

### Simulating Genetic Diversity

To explore the joint effects of heterogeneous mutation rates, stochastic genetic processes, and uncertainty in population history, we used the parameters inferred from the two-island and the isolation-migration coalescent models to simulate neutral genetic diversity in the program ms [62] (see Supporting Information, Table S3, for converting parameter estimates from lamarc and im to ms). We simulated 1,000 22-locus data sets, each consisting of 50 alleles per population to mimic our empirical sampling effort. To incorporate uncertainty in population history in the two-island model (Fig. 2a; Table 2), we randomly sampled 1,000 values for each demographic parameter from the joint posterior distributions from lamarc. This protocol resulted in 1,000 sampled histories, and we simulated data for all 22 loci under each history. In addition, we incorporated three other potential sources of among-locus heterogeneity in these simulations. (1) We incorporated differences in evolutionary rates among loci by sampling 1,000 independent estimates of *μ_R_* for each locus (selected every 10,000^th^ step) from the *beast analysis. In this case, we chose to sample steps, each of which contributed to the posterior distributions, rather than randomly sample directly from the posterior distributions because mean *μ_R_* for each simulated history must equal one by definition. Each set of *μ_R_* values was arbitrarily assigned to one of the sampled histories, and locus-specific values of *μ_R_* were used for each locus-specific simulation. (2) We included locus-specific recombination rates that were estimated from the lamarc analysis. To incorporate a variety of recombination rates, and hence uncertainty in those rates, we randomly sampled 1,000 rates for each locus from lamarc's posterior distributions. Locus-specific recombination rates were used for each locus-specific simulation. (3) Finally, we accounted for variance in fragment sizes among loci by multiplying *Θ* by the locus-specific fragment size for each simulation (Tables 1 & S3). Because CHD1Z is sex-linked, we adjusted parameters by a factor of 0.75 prior to conducting the simulations.

**Table 2:** Summary of the software, data, and parameters used to define in each of the five models simulated in this study.

			Model simulated		
	Two-island	Isolation-migration	Bottleneck	Hybridization	Selection
Demographic parameters^1^	lamarc: *θ_OW_*, *θ_NW_*, *M_OW_*, *M_NW_*	im: *θ_OW_*, *θ_NW_*, *θ_A_*, *M_OW_*, *M_NW_*, *t*, *s*	im: *θ_OW_*, *θ_NW_*, *θ_A_*, *M_OW_*, *M_NW_*, *t*, *s*	im: *θ_OW_*, *θ_NW_*, *θ_A_*, *M_OW_*, *M_NW_*, *t*, *s*	im: *θ_OW_*, *θ_NW_*, *θ_A_*, *M_OW_*, *M_NW_*, *t*, *s*
Number of loci	22	22	22	22	16
Recombination^1^	lamarc	lamarc	lamarc	lamarc	lamarc
Relative substitution rates (*μ_R_*)^1^	*beast	*beast	*beast	*beast	*beast
Additional parameters	-----	-----	lamarc: *θ_OW_* (i.e., *θ_pre-bottleneck_*)	im: *θ_falcated duck_*,*M_falcated duck→OW_*,*t_falcated duck-gadwall_*	-----

1 Incorporated uncertainty by sampling values from the posterior distributions calculated for each parameter.

To simulate genetic diversity under the isolation-migration model (Fig. 2b; Table 2), we chose parameter values from 1,000 histories (every 10,000^th^ step) visited during the Markov Chain in the im analysis. We converted *θ* for each locus by dividing *θ* by the geometric mean of fragment length among the loci and multiplying the resulting value by the locus-specific fragment size and *μ_R_* (sampled as described above). We also incorporated recombination rates from the lamarc analyses (as described above); in this way, we could address the full range of heterogeneity in our data by simulating genetic diversity over the full locus length rather than the truncated length. Thus, our simulations incorporated uncertainty in population history, uncertainty in relative substitution rates, uncertainty in recombination rates, and variance in fragment size (Table 2).

In addition to the basic two-island and isolation-migration models, we simulated data under three scenarios hypothesized to affect among-locus heterogeneity in genetic diversity (models are summarized in Table 2). First, we simulated a pre-divergence bottleneck. This model was a combination of the results from the isolation-migration model and the two-island model. We used the same 1,000 histories sampled for the isolation-migration model to define demographic parameters, but we assumed that the ancestral population had experienced a bottleneck prior to divergence. To define parameters associated with this bottleneck, we randomly selected values from a uniform distribution between *t* and 2*t* to vary the time of the bottleneck (*t_B_*) among the 1,000 simulated histories. For the period between time *t* and *t_B_* (pastwards in time), we defined population growth rates inferred from OW gadwalls (the probable ancestral population [10]) so that the population size continued shrinking (corresponding to an expansion forwards in time). At time *t_B_* the ancestral population instantaneously recovered (corresponding to a population crash forwards in time) to a size equal to *θ_OW_* estimated from lamarc; we used the same values of *θ_OW_* that were used in the two-island model, and each value was arbitrarily assigned to one of the 1,000 histories. In this way, we varied both the timing and the magnitude of the bottleneck among the 1,000 simulated datasets. We incorporated the three additional sources of heterogeneity (*μ_R_*, recombination, and fragment size) as described above.

Our second model considered the effects of gene flow from a third population (Table 2). Specifically, we simulated hybridization between gadwalls and their sister species, the falcated duck (*Anas falcata*). Hybridization between these taxa has resulted in mtDNA introgression into the gadwall gene pool, and there is also some evidence of CHD1Z introgression [50]. For these simulations, we used the results from the basic isolation-migration model, but incorporated migration rates obtained from Peters et al. [50]. Because that study only examined the mtDNA control region and two nuclear loci (LDHB and CHD1Z), the results were not directly comparable. However, in our ms simulations, we scaled all parameters to *θ_OW_* (see Table S3); thus, we were able to make the results comparable by scaling parameters estimated in Peters et al. [50] to *θ_OW_* from the same analysis. We sampled 1,000 estimates of *θ_fd_*/*θ_ow_* (size of the falcated duck population relative to the gadwall population), *θ_ow_M_ow_* (effective number of migrants from falcated ducks into OW gadwalls), *θ_ow_M_fd_* (effective number of migrants from OW gadwalls into falcated ducks scaled to *θ_ow_* as per ms guidelines), and *t*/*θ_ow_* (time since divergence scaled to the effective population size of OW gadwalls) from the posterior distributions. We assumed that any falcated ducks entering the NW population had to go through OW gadwalls, because these species are sympatric in Asia only—this scenario is consistent with the data [50]. Each set of values was then combined with one of the 1,000 histories sampled for the basic isolation-migration model, including the three additional sources of among-locus heterogeneity.

Our final model addressed the possibility that among-locus heterogeneity in selection has contributed to genetic diversity (Table 2). For this analysis, we first used the HKA software (available from Jody Hey, Rutgers University, Piscataway, NJ) to perform an HKA test [36] for selective neutrality. For this test, we compared the number of segregating sites in gadwalls to the average number of differences between gadwalls and each of the seven outgroup species. We then used an iterative process to determine which loci contributed significantly to overall deviations. Specifically, when an initial comparison showed significant deviations from the model, we removed the locus with the highest overall deviation and repeated the test. This was done for all 7 comparisons independently until each test was no longer significant. Loci that were eventually removed from more than 50% of the tests (*N*≥4 tests) were treated as *significant outliers*. We then repeated the isolation-migration analysis with the outliers excluded and simulated data with parameters drawn from those posterior distributions as described above for the basic isolation-migration model.

For each of the five simulated models, we calculated nucleotide diversity (*π*; OW and NW gadwalls combined), *Φ_st_*, and Tajima's *D* (averaged between OW and NW) from each locus (5 models×1,000 histories/model×22 loci/history = 110,000 simulated loci in total). These summary statistics were calculated using a script written in r [63] by TER (ms.out.r; datadryad.org; doi:10.5061/dryad.nv5v1v59). For each locus and model we generated posterior predictive distributions [64] of those summary statistics using the 1,000 locus-specific values. We also constructed posterior predictive distributions for both the means and coefficients of variation (a measure of heterogeneity) of *π*, *Φ_st_*, and Tajima's *D* calculated for each 22-locus dataset (1,000 values per model).

### Goodness-of-Fit Tests

We performed goodness-of-fit tests as described in Becquet and Przeworski [18]. We compared our empirical values of *π*, *Φ_st_*, and Tajima's *D* with the posterior predictive probabilities generated from the simulated datasets. For each comparison, we compared both the means and the coefficients of variation expected for a 22-locus dataset (1,000 replicates). We considered the test significant if the empirical values were within the 2.5% tails of the posterior predictive distributions (i.e., *P*≤0.05).

We also performed locus-specific goodness-of-fit tests [18] by applying the test to each locus separately. Here we compared the empirical value for each parameter with the posterior predictive probabilities generated with locus-specific parameters (fragment size, *μ_R_*, and recombination rates). Because one locus in a 22-locus dataset is expected to deviate significantly from the model by chance alone (with *α* = 0.05), we applied a correction for multiple tests based on the false discovery rate (FDR; [65]). We considered the test significant if the empirical values were within the 2.5% tails of the posterior predictive distributions after applying the FDR correction.

## Results

### Genetic Diversity and Population Structure

**Figure 3:**
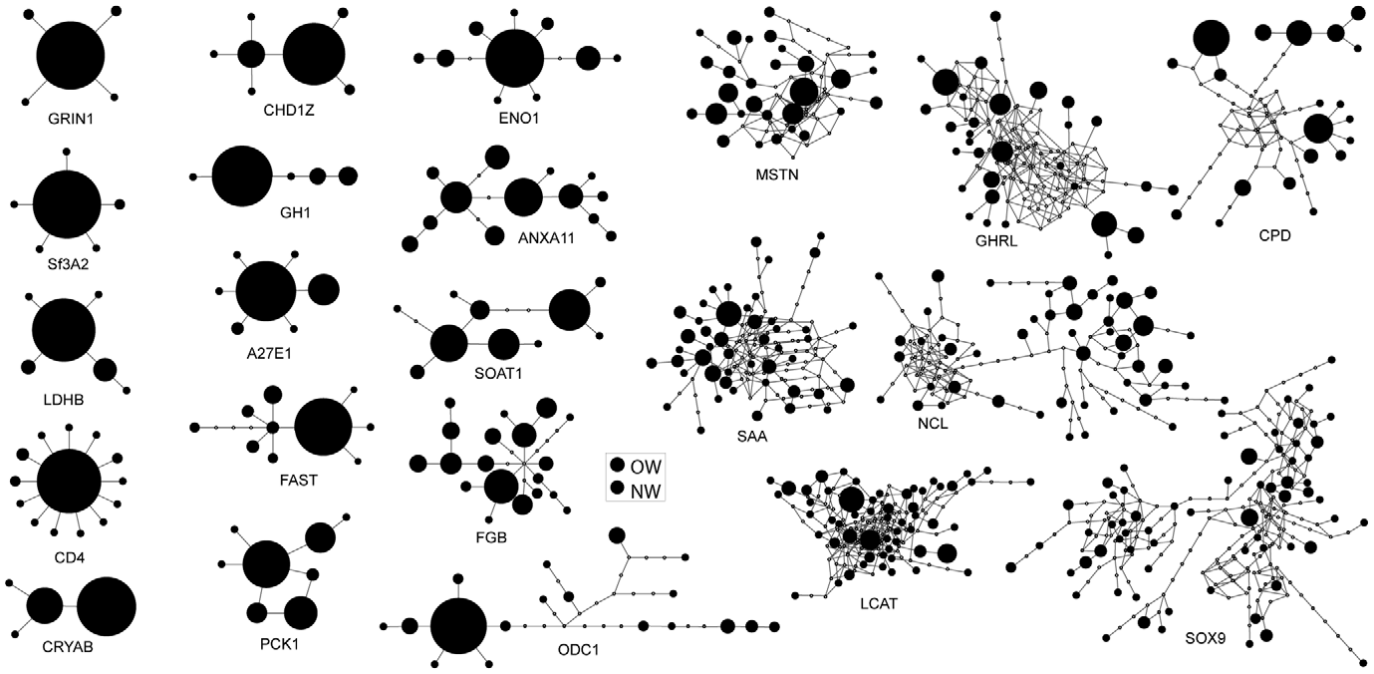
Haplotype networks. Haplotype networks illustrating the heterogeneity in genetic diversity among 22 non-coding loci sequenced from gadwalls. The area of the circles is proportional to the number of alleles occurring in the total sample (*N* = 50); substitutions are shown as branches between the alleles. See Table 1 for full gene names.

DNA sequences from 22 non-coding nuclear loci sequenced for 50 gadwalls revealed high heterogeneity in genetic diversity among loci (Fig. 3). Nucleotide diversity (*π*) varied across nearly two orders of magnitude (range = 0.0004 to 0.029; mean = 0.010±0.010 SD; Table 1), expected heterozygosity varied between 0.12 and 0.99 (mean = 0.62±0.30 SD), and allelic richness varied between five and 66 alleles per locus (mean = 20.0±18.6 SD). All three measures of genetic diversity were significantly correlated between OW and NW gadwalls (*R^2^*>0.86, *F*-ratio >58.7, *P*≤0.0000002), demonstrating that the heterogeneity was not specific to a single population.

structure indicated that the data best fit a two-population model (*K* = 2), with OW and NW gadwalls being genetically diagnosable (Fig. 1). In this model, 100% of OW gadwalls were assigned to population 1 with a mean assignment probability of 0.96 (±0.04 SD), and 100% of NW gadwalls were assigned to population 2 with a mean probability of 0.97 (±0.03 SD; Fig. 1). Only two individuals (both from Eurasia) received an assignment probability less than 95% (82.8% & 92.1%, respectively). Examining higher values of *K* and partitioning the data into separate OW and NW analyses failed to detect population structure within continents. Averaged across the 22 loci, 6.5% (mean *Φ_st_* = 0.065±0.075 SD) of the total genetic diversity was partitioned between OW and NW gadwalls (Table 1), and differences were significant at 14 loci (AMOVA, *P*≤0.05).

Mean Tajima's *D* was −0.59 (±0.87SD) and −0.16 (±0.87 SD) for OW and NW gadwalls, respectively. Tajima's *D* was significantly negative for four loci in OW gadwalls (A27E1, Sf3A2, CD4, and ENO1) and one locus in NW gadwalls (CD4), and values among loci were significantly correlated between OW and NW populations (*R^2^* = 0.46, *F*-ratio = 17.0, *P* = 0.0005). Averaging Tajima's *D* between the two populations, mean *D* was −0.37 (±0.80 SD; Table 1). Tajima's *D* was also significantly correlated with *π* in both populations (OW: *R^2^* = 0.44, *F*-ratio = 16.0, *P* = 0.0007; NW: *R^2^* = 0.34, *F*-ratio = 10.1, *P* = 0.005), indicating that low-diversity loci tended to have an excess of rare polymorphisms relative to high-diversity loci.

### Heterogeneous Substitution Rates

**Figure 4:**
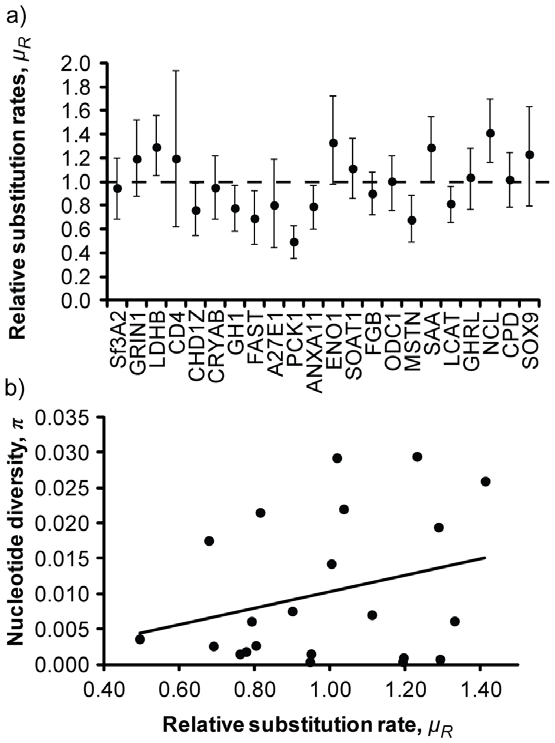
Substitution rates and genetic diversity. (a) Estimates of relative substitution rates (*μ_R_*) and their 95% higest posterior densities based on the analyses of eight taxa in *beast; loci are ranked on the x-axis (lowest to highest) by values of nucleotide diversity within gadwalls, and the horizontal dashed line indicates the mean relative rate (1.0 by definition). (b) Relationship between *μ_R_* and nucleotide diversity within gadwalls.

To test the hypothesis that heterogeneous substitution rates among loci caused the observed heterogeneity in genetic diversity, we estimated relative substitution rates (*μ_R_*) among the 22 loci using seven outgroup species. The 95% highest posterior distributions of *μ_R_* did not overlap for 38 pairs of loci, suggesting that substitution rates were significantly heterogeneous among loci (Fig. 4a). Overall, we found a 3-fold difference in *μ_R_* among loci (coefficient of variation, CV = 25%), which is similar to the 6-fold (CV = 32%) and 3-fold (CV = 21%) differences in evolutionary rates found in other large-scale studies of intron divergence in birds [66], [67]. However, this heterogeneity is low compared to the >75-fold difference observed in *π*, and *π* for gadwalls and *μ_R_* were not significantly correlated among loci (*R^2^* = 0.079, *F* = 1.72, *P* = 0.2; Fig. 4b), as predicted by neutral theory [36], [68]. Therefore, the observed differences in long-term substitution rates alone are insufficient for explaining the high among-locus heterogeneity that we found in genetic diversity.

Comparing intraspecific genetic diversity within gadwalls with interspecific divergence between gadwalls and each of the seven outgroup species revealed significant deviations from neutral expectations (HKA test; Sum of Deviations >50.1, df = 15–21, *P*<0.001, for all pairwise comparisons). Iteratively removing the loci that contributed the most to significant deviations required that 4–7 loci be removed before model expectations were met (i.e., the HKA test was non-significant). For all seven outgroup comparisons, LDHB uniformly had the highest deviation (Supporting Information; Fig. S1). Iteratively removing one additional locus at a time, CRYAB and GH1 also contributed to strong deviations and were ultimately removed from each test. GRIN1 and Sf3A2 were iteratively removed from five and four of the tests, respectively. Finally, SOAT1, CHD1Zb, and FGB contributed to significant deviations in one or two of the models each. All seven loci had a paucity of segregating sites within gadwalls relative to interspecific divergence.

### Population History

**Figure 5:**
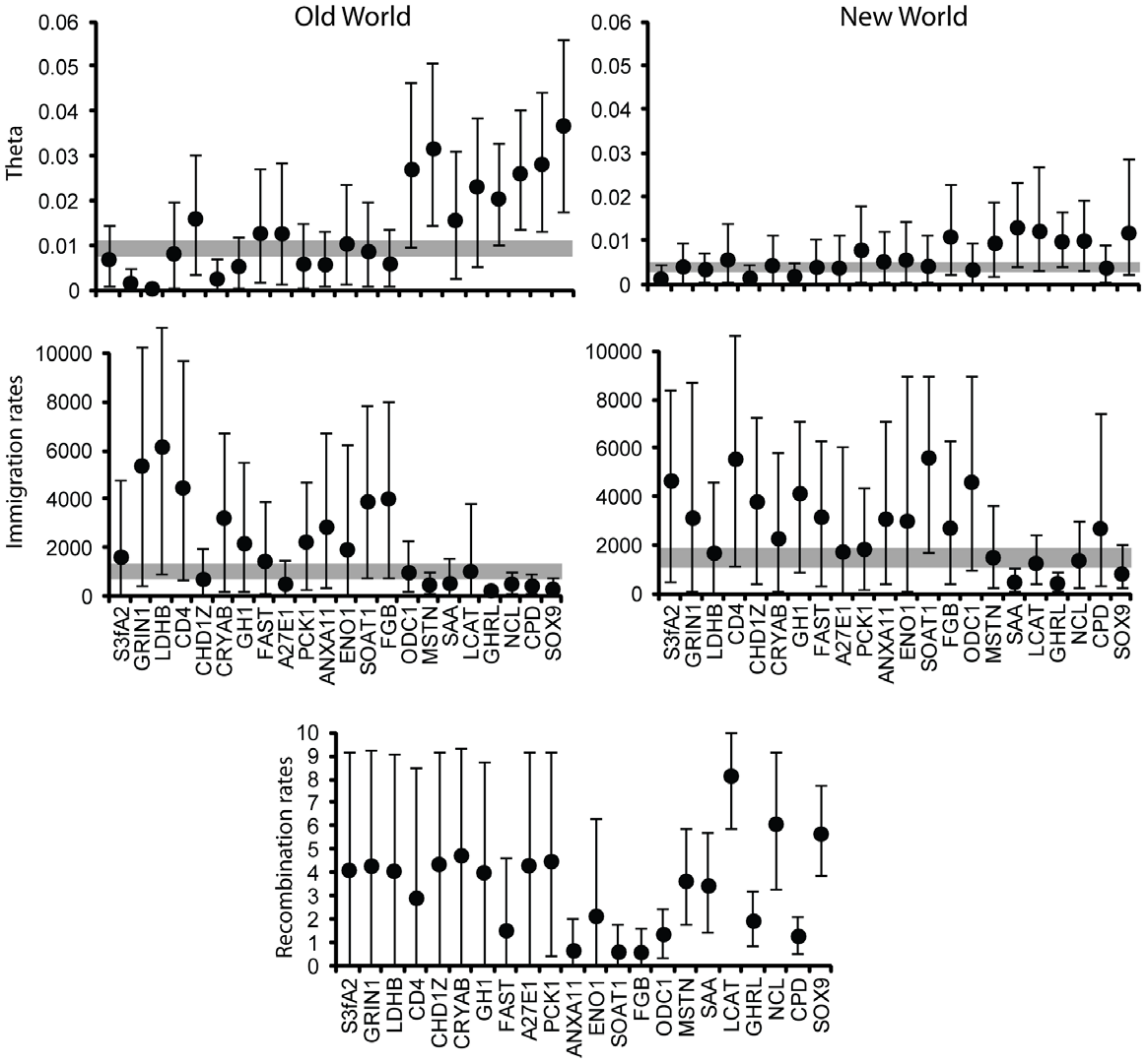
Two-island model results. Mean and 95% HPDs of estimates of the five parameters from the two-island model of population divergence. Gray shading indicates the joint estimates obtained by multiplying the posteriors among loci. Loci are ranked by nucleotide diversity from low to high.

The two-island model of population divergence suggested high heterogeneity in *Θ* among the 22 loci, even after controlling for heterogeneous substitution rates (including uncertainty in *μ_R_*; Fig. 5). The 95% highest posterior distributions (HPDs) did not overlap for 35 pairs of loci for *Θ_OW_*, but overlapped between all pairs for *Θ_NW_*. Calculating joint estimates of *Θ* resulted in a narrow range of values that were consistent with the observed genetic diversity at all loci for both OW (*Θ* = 0.0092, 95% HPD = 0.0077–0.011) and NW (*Θ* = 0.0042, 95% HPD = 0.0028–0.0052) populations (Fig. 5). Regardless, 17 loci and the joint estimates supported higher effective population sizes for OW gadwalls relative to NW gadwalls. Estimates of *M* among loci were less heterogeneous, with 7 and 6 pairs of loci having non-overlapping 95% HPDs for *M_OW_* and *M_NW_*, respectively (Fig. 5). Joint estimates of *M* suggested higher gene flow (forward in time) into North America (*M_NW_* = 1480, 95% = 1050–1850) than into Eurasia (*M_OW_* = 1010, 95% CI = 660–1340). Recombination rates also varied significantly among loci, with higher-diversity loci tending to have higher recombination rates, although low-diversity loci contained little information regarding recombination (Fig. 5).

**Figure 6:**
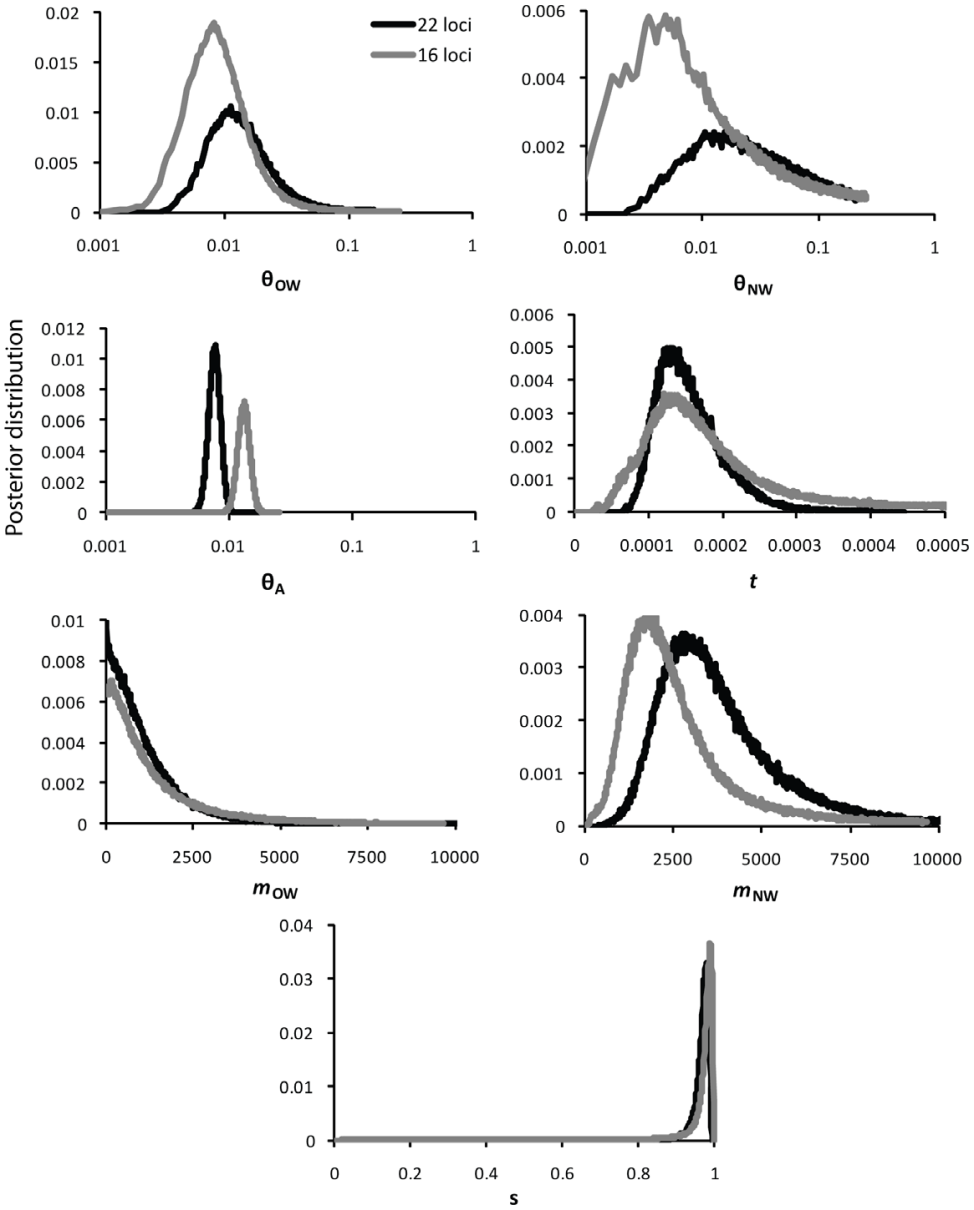
Isolation-migration model results. Posterior distributions of the seven parameters estimated using the isolation-migration model of population divergence. Heavy lines are the posterior distributions from the analysis of the full 22-locus dataset; light lines are from the analysis of the 16-locus dataset excluding six loci that may be under selection. Values are rescaled to the per-site substitution rate.

Inferring an isolation-migration model in IM, all parameters had finite posterior distributions except *θ_NW_*, which contained a flat tail (Fig. 6). In this model, *θ_ow_* and *θ_nw_* did not differ (*θ_ow_* = 2.53, 95% HPD = 0.83–23.4; *θ_nw_* = 2.98, 95% HPD = ∼0.63–44.8), but *θ_a_* was generally smaller and had a narrower confidence interval (*θ_a_* = 1.69, 95% HPD = 1.36–2.13). The splitting parameter, *s*, suggested that only 2.2% (95% HPD = 0.7–8.3%) of the ancestral population contributed to the NW population at the time of divergence (*t* = 0.032, 95% HPD = 0.016–0.059). Consistent with the two-island model, the isolation-migration model supported asymmetrical gene flow with higher rates (forward in time) into North America (*M*_nw_ = 12.2, 95% HPD = 4.8–33.0) than into Eurasia (*M*_ow_ = 0.13, 95% HPD = ∼0–13.1). Overall, these results from 22 loci were consistent with results from a smaller dataset that included five introns and the mtDNA control region [10].

### Simulations

**Figure 7:**
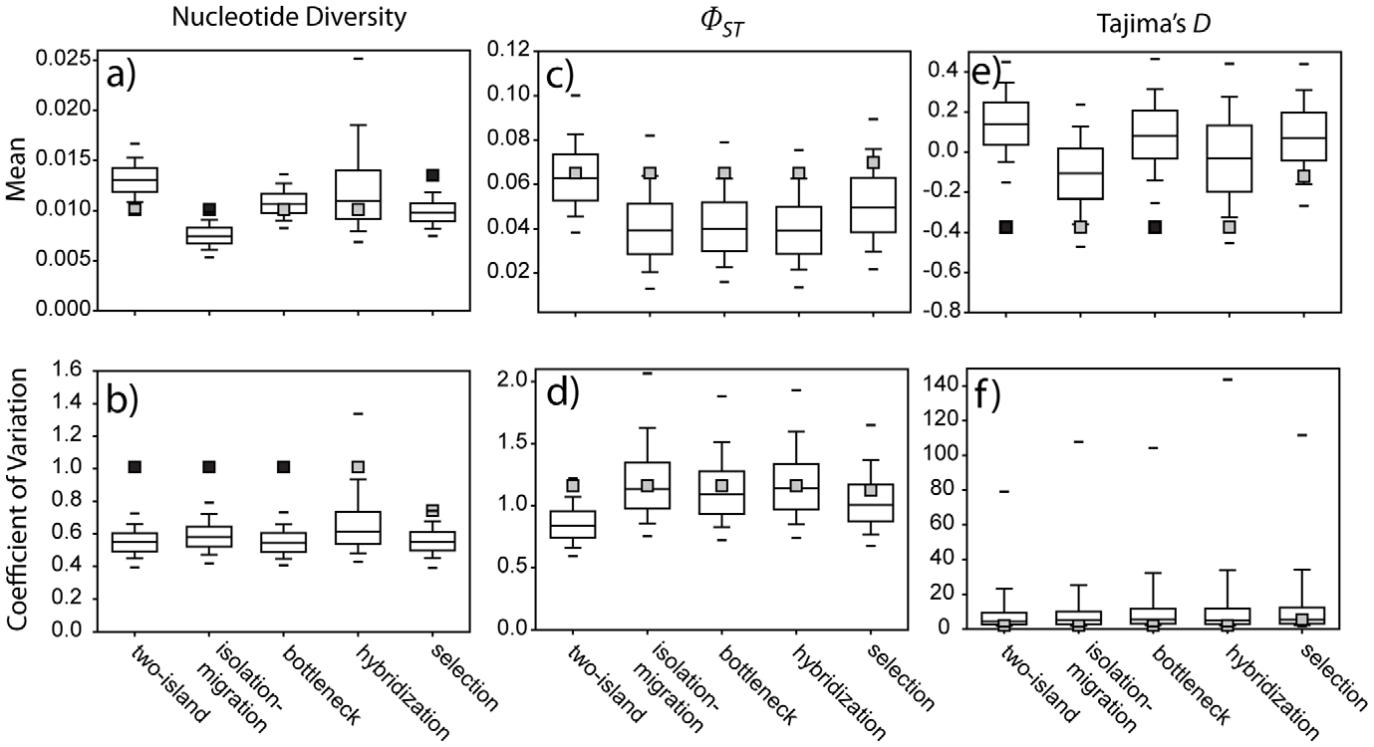
Goodness-of-fit tests of five models of population history. Box plots indicate the posterior predictive distributions of the mean (a, c, e) and coefficient of variation (b, d, f) for nucleotide diversity (a, b), *Φ_st_* (c, d), and Tajima's *D* (e, f) simulated for a 22-locus dataset (or a 16-locus dataset in the selection model) with 1,000 replicates; horizontal lines indicate the 95% confidence limits. Lightly shaded squares mark the values for empirical data that fell within the 95% confidence intervals; dark shading indicates empirical values that fell within the 2.5% tails of the posterior predictive distributions.

To test for the combined effects of heterogeneous substitution rates and the stochastic variance of genetic processes, we simulated DNA sequences under selective neutrality using the parameters estimated from the two models of population divergence (Fig. 2). Simulations under the two-island model over-predicted mean *π*, whereas simulations under the isolation-migration model under-predicted mean *π* (Fig. 7a); however, only the deviation from the isolation-migration model was significant (*P* = 0.016). Furthermore, the dispersion of values around the mean (coefficients of variation, CVs) was significantly higher than expected for both models (*P* = 0.001; Fig. 7b). In contrast, values of *Φ_st_* (both mean and CV) were within the 95% CIs for both models (Fig. 7c–d). Simulations under the two-island model, but not the isolation-migration model, significantly over-predicted Tajima's *D* (*P*<0.001; Fig. 7e). The CVs for *D* were within the CIs for both models (Fig. 7f).

**Figure 8:**
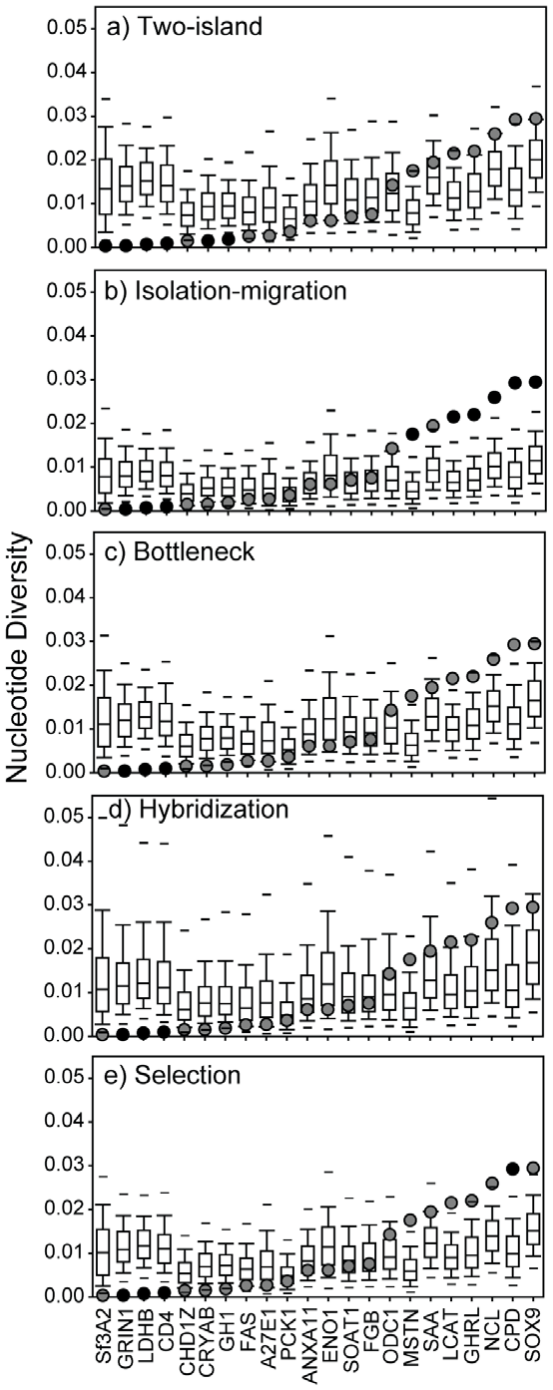
Goodness-of-fit tests of locus-specific nucleotide diversity from five models of population history. Box plots indicate the posterior predictive distributions for each locus (1,000 replicates; horizontal lines indicate the 95% confidence limits). Light-shaded circles mark the values for empirical data that fell within the 95% confidence intervals, whereas dark-shaded circles mark significant outliers (after applying a correction for the false discovery rate). GRIN1, LDHB, and CD4 consistently deviated from the simualted values. Loci are ranked on the x-axis by nucleotide diversity.

Locus-specific goodness-of-fit tests revealed that six loci (Sf3A2, GRIN1, LDHB, CD4, CRYAB, and GH1) had significantly lower *π* than expected under the two-island model (Fig. 8a). Under the isolation-migration model, three low-diversity (GRIN1, LDHB, and CD4) and six high-diversity loci (MSTN, LCAT, GHRL, NCL, CPD, and SOX9) had values of *π* that deviated significantly from the simulated values (Fig. 8b). Thus, values of *π* deviated from the models for 27.3% and 40.9% of the loci examined. Likewise, one locus (CD4) had a significantly more negative value for Tajima's *D* in both models, but all values of *Φ_st_* were within the 95% CIs of the posterior predictive distributions. Regardless of the differences between the two models, both demonstrated that the combined effects of stochastic processes and heterogeneous substitution rates cannot fully account for the high heterogeneity we observed in *π*.

Our method for defining a bottleneck resulted in population sizes changing by −97% to 43% (mean = −56%; 95% CI = −15% to −91%) from the long-term ancestral size to the bottlenecked population size (positive values of population size change resulted from six histories in which the ancestral *N_e_* inferred from im was larger than the long-term *N_e_* inferred from lamarc). Simulating a pre-divergence bottleneck resulted in higher mean values of *π* compared to the basic isolation-migration model (Fig. 7a) but not the CV (Fig. 7b). Indeed, the empirical CV was significantly higher than simulated values (*P*<0.001). Furthermore, the simulated CV was not related to the magnitude of the bottleneck (*R* ^2^ = 0.0002, df = 999, *F*-ratio = 0.18, *P* = 0.67). Empirical values of mean *Φ_st_* and the associated CV were within the 95% CIs of the simulated data (Fig. 7c, d). Similar to the basic two-island model, the bottleneck model significantly over-predicted mean Tajima's *D* (*P* = 0.004; Fig. 7e), but the empirical CV fell within the 95% confidence intervals of the simulated data (Fig. 7f).

Relative to the basic models, the locus-specific values of *π* were more consistent with the bottleneck model, with only three loci (GRIN1, LDHB, and CD4; all low-diversity) significantly deviating from the simulated values (Fig. 8c). All locus-specific values of *Φ_st_* were within the 95% CIs, and Tajima's *D* deviated from the simulated values only for CD4.

Incorporating introgression from a third population (i.e., hybridization with the falcated duck) had the largest effect on the mean and CVs for *π* (Fig. 7a, b). We found both higher means and higher CVs under this model, and the empirical values were within the 95% CIs for both measures. However, GRIN1, LDHB, and CD4 continued to have lower diversity than simulated data (Fig. 8d). Mean and CVs for Tajima's *D* and *Φ_st_* were all within the simulated range of values (Fig. 7c–f), and only CD4 had a significantly negative *D*.

On the basis of the HKA test, we excluded Sf3A2, GRIN1, CRYAB, LDHB, and GH1 from analyses to address the possibility that selection has contributed to the among-locus heterogeneity that we observed. We also excluded CD4 from this analysis, because this locus consistently had a paucity of *π* and a more negative value for Tajima's *D* in previous models. Removing these six loci resulted in smaller estimates of *θ_nw_* and *M*_nw_, but estimates of *θ_ow_*, *M*_ow_, *t*, and *s* did not change appreciably (Fig. 6). The most prominent difference between this selection model and the basic isolation-migration model was that *θ_A_* was significantly larger after removing loci inferred to be under selection (Fig. 6). Compared to the basic model, simulating data under the selection model resulted in a better fit between mean *π* for the 16-locus dataset and model expectations, although *π* was still slightly under-predicted (*P* = 0.012; Fig. 7a). However, the CV for the 16-locus dataset was within the 95% CIs of the posterior predictive distributions (Fig. 7a, b). Furthermore, empirical values of *π* for 15 of the 16 loci were within the 95% CIs (CPD had higher diversity than expected; Fig. 8e); GRIN1, LDHB, and CD4 continued to deviate from expectations. Results for Tajima's *D* and *Φ_st_* were consistent with the above analyses.

## Discussion

Sequences from 22 non-coding, nuclear loci in Holarctic gadwalls revealed high among-locus heterogeneity in genetic diversity, and this heterogeneity did not fit simple models of neutral population histories. The two-island model moderately over-predicted mean values of *π*, whereas the isolation-migration model under-predicted *π*. Furthermore, the observed among-locus heterogeneity was significantly higher than expected under both neutral models. Because we incorporated relative substitution rates obtained from outgroup comparisons, heterogeneous substitution rates alone cannot explain the among-locus heterogeneity that we observed. Likewise, our use of allele-specific priming to resolve the gametic phases of alleles confirmed that our results were not an artifact of amplifying and sequencing paralogs [48]. Thus, the observed heterogeneity suggests that our data violate key assumptions of the models, and that these violations likely bias estimates of population history. We will now examine some of these assumptions.

### Changes in Population Size

The two-island model assumes that *N_e_* has been constant over time. In contrast, the isolation-migration model assumes exponential size changes following divergence, but that the ancestral *N_e_* has been constant. Any other changes in population sizes would violate these assumptions and could have contributed to the poor fit between the empirical data and the models. For example, bottlenecks of moderate strength can cause high among-locus heterogeneity in *π*, which can result in an overly liberal HKA test [28], [30]. However, including a pre-divergence bottleneck in our simulations did not appreciably change the variance expected under the isolation-migration model, despite simulating data using a broad range of values for both the timing and the magnitude of the simulated bottleneck. Furthermore, we did not find a significant relationship between the among-locus heterogeneity in *π* and the magnitude of the simulated bottleneck. Although there are an infinite number of possible bottleneck scenarios that have not been examined here, a pre-divergence bottleneck seems insufficient for explaining the high among-locus heterogeneity in our empirical dataset [27], [28], [31].

Long term fluctuations in population sizes, which we did not explicitly examine, could also have contributed to our findings. Fluctuations in population size cause *N_e_* to be approximately equal to the harmonic mean of long-term population size [69], [70]. Because *Θ* is a function of *N_e_*, an assumption of constant size would seem adequate. However, when using genetic data, *Θ* is estimated over the genealogy and thus represents the harmonic mean of *N_e_* between the present and the time of the most recent common ancestor (TMRCA) within the sampled genealogy. Given the differences in nucleotide diversity among our loci, TMRCA likely varied considerably, and this variance could result in among-locus heterogeneity in *Θ*. For example, if population sizes were small in the recent past, then any locus that coalesces within that timeframe would have a small *Θ*. However, a locus with a substantially older TMRCA could include periods of larger sizes within their history, which would cause *Θ* to be larger. Thus, fluctuating population sizes contributing to among-locus differences in TMRCA theoretically could have caused the high among-locus heterogeneity in *Θ* that we observed in the two-island model. Despite allowing for exponential growth or decline following divergence, the isolation-migration model could also be sensitive to among-locus differences in timescales reflected in our data, because this model assumes a constant ancestral *N_e_*. This possibility is supported by our observation that removing the low-diversity loci (those inferred to be under selection) from the im analysis resulted in a significantly larger estimate for the ancestral population size and a better fit between the empirical and the simulated data.

### Hybridization and Gene Flow

Both the two-island and the isolation-migration model assume that the sampled populations do not exchange genes with other unsampled populations. Ducks are well known for their capacity to hybridize and produce fertile offspring with other species [71]–[74], and larger sample sizes of gadwalls revealed introgression of mtDNA from several species [38], [50]. In particular, about 5% of North American gadwalls carry mtDNA haplotypes derived from falcated ducks, and one Asian gadwall had a putatively introgressed CHD1Z allele (no evidence of introgression for LDHB was found). Thus, falcated ducks and other species potentially contributed to the nuclear gene pool of gadwalls as well, which could have caused heterogeneity among loci. In support of this hypothesis, we found that incorporating hybridization from falcated ducks into our simulations resulted in the CV for *π* to be consistent with the observed empirical data. These simulations demonstrate that the stochasticity of genetic drift can cause the genetic contribution of a third population to vary among loci, thus creating among-locus heterogeneity in genetic diversity.

Although hybridization is a strong candidate for explaining our results in gadwall, results from a previously published simulation study [24] seem inconsistent with this hypothesis. Specifically, gene flow with a third population tends to cause ancestral population sizes to be overestimated and to have large CIs [24]. In contrast, our isolation-migration results suggested that the ancestral population size was small relative to current sizes and the estimate had a narrow CI. The effects of interspecific hybridization warrant further study, especially using an *n*-population model [75] that includes sequences from falcated ducks.

### Selection

Both im and lamarc assume that the loci studied are selectively neutral. However, selection can affect polymorphisms in non-coding DNA both directly and indirectly. For example, components of introns such as structural and regulatory elements are functional and selectively constrained [76], [77]. Indirect effects of selection via genetic hitchhiking can also alter genetic signatures in non-coding DNA that is closely linked to coding exons [78], [79]. Indeed, there is growing evidence that selection can have a prominent effect on polymorphisms in non-coding DNA [80]–[88]. Although different forms of selection can create patterns that mimic the genetic signatures of various population histories [15], the overall importance of selection in biasing inferences of population-level parameters is not well understood.

Three lines of evidence support the hypothesis that selection has influenced some of our loci. First, low-diversity loci were more likely than high-diversity loci to contain an excess of rare polymorphisms, which is consistent with the effects of purifying or directional selection acting at those loci [89]. For example, CD4 is critical for an adaptive immune response and has a conserved interaction with the class II major histocompatibility complex that is required for the activation of T-helper cells [90]–[92]. Accordingly, the CD4 gene is likely subject to strong selection, which could have an indirect effect on polymorphisms within the linked introns. Consistent with this possibility, CD4 had low nucleotide diversity and an excess of rare polymorphisms (i.e., a significantly negative Tajima's *D*) relative to the values simulated under all five of our models. Furthermore, the network topology exhibited the classic star-like pattern (Fig. 3) suggestive of a selective sweep [34]. GRIN1, Sf3A2, and LDHB also exhibited this star-like network, negative Tajima's *D*s, and a paucity of intraspecific polymorphisms relative to interspecific divergence, all of which are consistent with selective sweeps. Second, removing low-diversity loci that the HKA test detected as significant outliers resulted in a better fit between the heterogeneity observed in the empirical data and data simulated under the isolation-migration model. Third, removing the low-diversity loci resulted in a significantly larger estimate of the ancestral *N_e_*, suggesting that different categories of loci contain heterogeneous signatures of population history. This heterogeneity is also reflected in the among-locus differences in *Θ* estimated from the two-island model. Although the HKA test might have caused the liberal removal of loci (i.e., loci not influenced by selection; see [28]), these results demonstrate that selection is a strong candidate for explaining the among-locus heterogeneity in *π* that we observed.

### Population Structure

Both models assume that the populations are each panmictic. This assumption seems reasonable for our data. First, structure analyses best supported a two-population model (OW and NW), and repeating the analyses for each continent separately did not detect any additional structure. Second, a larger sample size of individuals for three nuclear loci revealed that genetic variation was significantly partitioned between OW and NW populations, but not among sampling localities within continents [10]. Furthermore, Strasburg and Rieseberg [24] found that im was generally insensitive to even moderate levels of population substructure. Thus, it is unlikely that undetected substructure within our OW and NW populations explains the deviations from the models of population history. Structure within the ancestral population is also unlikely to explain our results, because this violation should have resulted in a large ancestral population size [23], which we did not find.

### Population History and Basic Model Differences

In addition to finding a poor fit between the empirical data and the basic coalescent models, we found that simulating data under the inferred two-island and isolation-migration models gave different null expectations, especially for *π* and Tajima's *D*. One possible explanation for these discrepancies was the manner in which recombining loci were handled. Whereas lamarc incorporates recombination into the analyses, im assumes no recombination. To meet this assumption of no recombination, we used a recombination-filtered data set that removed 19.4% of the nucleotides and 41.6% of the segregating sites from the im analysis. Simulations show that this practice of truncating sequences causes a systematic downward bias in estimates of *θ* [24], [61]. This bias might have been especially problematic in our data set, because only small fragments of high-diversity loci could be used, whereas the low-diversity loci did not require truncating. If using recombination-filtered data sets caused im to underestimate *θ*, then mean *π* also would be under-predicted in our simulations, as we observed for the isolation-migration model. However, this difference cannot explain why the two-island model over-predicted mean *π*.

Other differences between the models could also have contributed to the contrasting results. The isolation-migration model included estimates of divergence time, ancestral population size, and population growth rates, which were not incorporated into the two-island model. Indeed, assuming a constant *N_e_* in the two-island model is a probable explanation for the over-prediction of Tajima's *D* in the simulated data. In addition, im infers differences in substitution rates (mutation scalars) from the data analyzed [20], whereas we defined relative substitution rates for the lamarc analysis that were estimated from independent data. Any differences in the inferred rates could have contributed to differences between parameters estimated from the two models, especially for *θ* and *π*. Despite these inconsistencies, it is encouraging that both models supported a larger *N_e_* for OW gadwalls relative to NW gadwalls (average *θ* over the long term), and both models supported asymmetrical gene flow, with greater movements from OW to NW than vice versa.

### Conclusions

The high heterogeneity in nucleotide diversity that we observed among 22 non-coding loci in gadwall ducks did not fit simple, neutral models of population history. Based on simulations, interspecific hybridization and selection are both strong candidates for causing the observed deviations from the models. The effects of hybridization and selection could be synergistic, thereby having an additive effect on among-locus heterogeneity. For example, selection could inhibit or prevent some genes from crossing species or population boundaries, which can create heterogeneous patterns among different loci [8], [32], [43], [44], [93]. More specifically, loci with a higher propensity for introgression would have a higher *N_e_* than loci for which gene flow is restricted. Examining both of these hypotheses simultaneously might provide a better understanding of the complexity underlying genetic diversity within the genomes of diverging populations.

Given our results suggesting that genomic diversity is more complex than predicted by available coalescent models, one might question the value of these methods for studying population histories, especially given their sensitivity to the violation of assumptions [23], [24]. We argue that our results do not undermine the value of coalescent models but rather demonstrate the need to test how well empirical data fit these models. The results from coalescent analyses serve as an invaluable null model, and comparing empirical and simulated data enables the evaluation of factors that might have contributed to a lack of fit. Furthermore, other research might show that sequence data from other species and populations fit the models well. In either case, coalescent methods coupled with coalescent simulations offer rigorous means of examining how historical events have contributed to DNA polymorphisms, and thus should continue to provide insights into the generation and maintenance of genetic diversity.

## Supporting Information

Figure S1 **HKA test.** Deviation of measures of genetic diversity calculated by comparing 22 loci between gadwall and each of seven outgroup species using an HKA test.
(TIF)

Table S1 **Primers used for amplifying 22 non-coding loci in gadwalls.**
(DOCX)

Table S2 **List of voucher specimens and GenBank accession numbers for previously published sequences used in this study.**
(DOCX)

Table S3 **Equations for converting parameters estimated in im and lamarc to the appropriate scale for simulating genetic diversity in the program ms.**
(DOCX)
