# Supplementary figures and images for: Heterogeneity in Genetic Diversity among Non-Coding Loci Fails to Fit Neutral Coalescent Models of Population History
Source: PLoS One. 2012 Feb 22;7(2):e31972. doi: 10.1371/journal.pone.0031972 (PMC3285185; doi:10.1371/journal.pone.0031972)

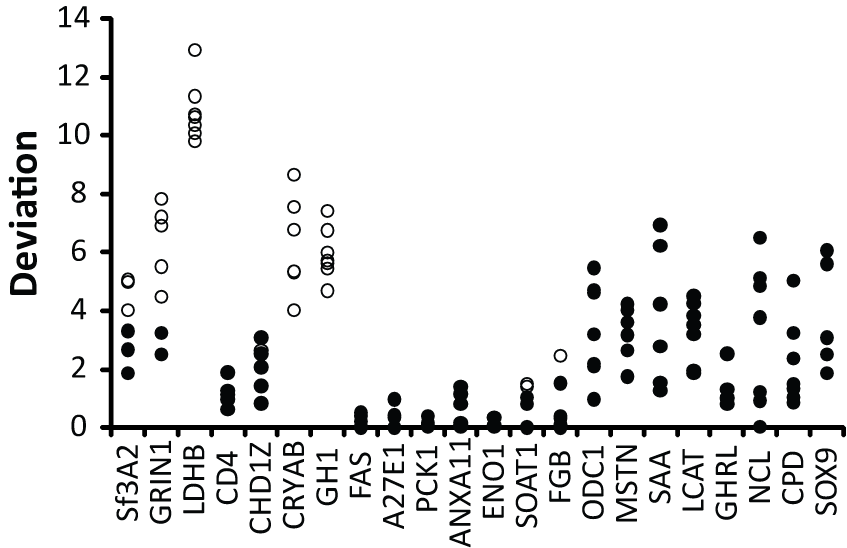

Supplement: Figure S1 — HKA test. Deviation of measures of genetic diversity calculated by comparing 22 loci between gadwall and each of seven outgroup species using an HKA test. (TIF) [file pone.0031972.s001.tif]
