# Supplementary material for: Heterogeneity in Genetic Diversity among Non-Coding Loci Fails to Fit Neutral Coalescent Models of Population History
Source: PLoS One. 2012 Feb 22;7(2):e31972. doi: 10.1371/journal.pone.0031972 (PMC3285185; doi:10.1371/journal.pone.0031972)
Supplement: Table S1 — Primers used for amplifying 22 non-coding loci in gadwalls. (DOCX) [file pone.0031972.s002.docx]

Table S1. Primers used for amplifying 22 non-coding loci in gadwalls.

| Locus | Primers |
| --- | --- |
| CHD1Z | F: 5'-CAGAGAATGGACACGACAGG-3'  R: 5'-AGTTCCTCTGCACCAAACTTTA-3' |
| LDHB | F: 5'-ATGGGAGCTTGTTCCTTCAA-3'  R: 5'-CTTCCTGCTGACGAACACCT-3' |
| CD4 | F: 5'-CTCCATCGATTAATNAGAACATCTCC-3'  R: 5'-TTCCKGAAGTTCAGAYGCCATGAC-3' |
| FAST | F: 5'-CTGGTAATATTTGTGTCAGTGCTTA-3'  R: 5'-AAGATCCAAAACTGCAGCAA-3' |
| ODC1 | F: 5'-TCGTTCAAGCCATTTCTGATGCC-3'  R: 5'-CCAGGRAAGCCACCACCAATRTC-3' |
| FGB | F: 5'-GTTAGCATTATGAACTGCAAGTAATTG-3'  R: 5'-TTTCTTGAATCTGTAGTTAACCTGATG-3' |
| SAA | F: 5'-GCAGCCAGAGTGATCAGGTA-3'  R: 5'-GTTCCTCACTCGCCTAACG-3' |
| ANXA11 | F: 5'-AGTSTTGAGGAAGGCCATGA-3'  R: 5'-GCTTGTTTGAACGACTTCCA-3' |
| MSTN | F: 5'-CACCCTCAAAATTTCTGCAAC-3'  R: 5'-GATGCTAGCACAAAGATGATGG-3' |
| SOAT1 | F: 5'-CCTCTGCATCTTCAACTCCA-3'  R: 5'-GAAGGCAAAGAAGACCAGGA-3' |
| NCL | F: 5'-CGCTAAGAGAATCATTTGAAGG-3'  R: 5'-TCTTCTGGGGAGCTGAAGTC-3' |
| GHR | F: 5'-GGCAACGACRGTATTGAACT-3'  R: 5'-TGATGCACAAAAGGTATAAGAATGT-3' |
| GRIN1 | F: 5'-CTGGTGGGGCTGTCTGTG-3'  R: 5'-ACTTTGAASCGKCCAAATG-3' |
| SOX9 | F: 5'-CTCCAGCATCAGCGAGGT-3'  R: 5'-GGGGTAACACAAACAGCTC-3' |
| CPD | F: 5'-GATGGGTACCAACARAAGCA-3'  R: 5'-GAACACAGAGCTGGGTGCAT-3' |
| PCK1 | F: 5'-CAGCCATGAGATCTGAAGCA-3'  R: 5'-TTGAGAGCTGGCTTTCATTG-3' |
| ENO1 | F: 5'-CGCGATGGAAAGTATGACCT-3'  R: 5'-CCAACGCTGCCAGTAAACTT-3' |
| CRYAB | F: 5'-CAGCCCCTTCCTGATGAGAT-3'  R: 5'-AAAGCTTTGCAGGAAATTCG-3' |
| GH1 | F: 5'-CTCAGAAACCATCCCAGCTC-3'  R: 5'-GAGAACCAGTGAAAACCGAAG-3' |
| AMH | F: 5'-CTGGAACAGGGAAACCAAAC-3'  R: 5'-GGAGGACGCAGTGTTGTGTA-3' |
| LCAT | F: 5'-CTCAACACATTCCTGCCAGT-3'  R: 5'-GACATTTTCCGGGAGGTTC-3' |
| A27E1 | F: 5'-ACATCTTTGGCATTTTGAA-3'  R: 5'-CATCCACTAGAACACAGACATT-3' |
