# Supplementary material for: Heterogeneity in Genetic Diversity among Non-Coding Loci Fails to Fit Neutral Coalescent Models of Population History
Source: PLoS One. 2012 Feb 22;7(2):e31972. doi: 10.1371/journal.pone.0031972 (PMC3285185; doi:10.1371/journal.pone.0031972)
Supplement: Table S2 — List of voucher specimens and GenBank accession numbers for previously published sequences used in this study. (DOCX) [file pone.0031972.s003.docx]

Table S2. List of voucher specimens and genbank accession numbers for previously published sequences used in this study.

| Species | Voucher | CHD1Z | LDHB | ENO1 | ODC1 | A27E1 | FGB | PCK1 | GH1 |
| --- | --- | --- | --- | --- | --- | --- | --- | --- | --- |
| *Anas strepera*^1^  AB0345 |  | EU555667 | EU555816 | JQ255606 | EU555945 | JQ180638 | ----- | ----- | ----- |
| AK8012 |  | EU555676 | EU555821 | EU556121 | EU555948 | EU556062 | ----- | ----- | ----- |
| AK1501 |  | AY881937 | EF537841 | EU556120 | EU555949 | EU556061 | ----- | ----- | ----- |
| AK4611 |  | EU555686 | EU555827 | EU556122 | EU555955 | EU556063 | ----- | ----- | ----- |
| AR4 |  | EU555694 | EU555832 | EU556124 | EU555963 | EU556065 | ----- | ----- | ----- |
| CO4369 |  | AY881947 | EF537834 | EU556129 | EU555965 | EU556070 | ----- | ----- | ----- |
| DE82 | DMNH 83682 | EU55571 | EU555846 | EU556130 | EU555966 | EU556071 | ----- | ----- | ----- |
| KL23 |  | EU555697 | EU555837 | EU556127 | EU555968 | EU556068 | ----- | ----- | ----- |
| MB5 |  | EU555720 | EU555851 | EU556131 | EU555980 | EU556072 | ----- | ----- | ----- |
| MD9 |  | EU555729 | EU555858 | EU556132 | EU555987 | EU556073 | ----- | ----- | ----- |
| MT4760 |  | AY881948,AY881949 | EF537835 | EU556134 | EU555988 | EU556075 | ----- | ----- | ----- |
| NB3 |  | EF537769 | EF537827 | EU556135 | EU555989 | EU556076 | ----- | ----- | ----- |
| ND002 |  | EU555737 | EU555864 | EU556136,EU624484 | EU555991,EU624473 | EU556077 | ----- | ----- | ----- |
| ND013 |  | EU555741 | EU555865 | EU556137 | EU555995 | EU556078 | ----- | ----- | ----- |
| NV7821 |  | AY881954,AY881955 | EF537837 | EU556138 | EU556001 | EU556079 | ----- | ----- | ----- |
| NY6935 |  | AY881953 | EU555869 | EU556139,EU624485 | EU556002 | EU556080 | ----- | ----- | ----- |
| OR9029 |  | EF537771 | EF537839 | EU556140 | EU556003 | EU556081 | ----- | ----- | ----- |
| QC0081 |  | EF537772 | JQ255607 | EU556141 | EU556006,EU624475 | EU556082 | ----- | ----- | ----- |
| SD5319 |  | AY881950 | EF537830 | EU556142 | EU556012 | EU556083 | ----- | ----- | ----- |
| SK10 |  | EF537775 | EU555875 | EU556144,EU624486 | EU556013 | EU556085 | ----- | ----- | ----- |
| SK0296 |  | EU555749 | EF537831 | EU556143 | EU556016,EU624476 | EU556084 | ----- | ----- | ----- |
| SM62 |  | EU555714 | EU555845 | EU556128 | EU555977 | EU556069 | ----- | ----- | ----- |
| UT1714 | UWBM 70833 | AY881938 | EF537836 | EU556145 | EU556022 | EU556086 | ----- | ----- | ----- |
| WA8 |  | EU555772 | EU555887 | EU556147 | EU556027 | EU556087 | ----- | ----- | ----- |
| WY3889 |  | AY881946 | EF537833 | EU556148 | EU556028 | EU556088 | ----- | ----- | ----- |
| 18797 | UAM 18797 | EU555620 | EU555774 | EU556089 | EU555888 | EU556029 | ----- | ----- | ----- |
| B17247 | LSUMZ B17247 | EU555621 | EU555775 | EU556090 | EU555889 | EU556030 | ----- | ----- | ----- |
| CZ4 |  | EU555626 | EU555778 | EU556095 | EU555890,EU624450 | EU556035 | ----- | ----- | ----- |
| CZ5 |  | EU555627 | EU555779 | EU556096 | EU555891 | EU556036 | ----- | ----- | ----- |
| FEB89 |  | EF537739 | EF537822 | EU556092 | EU555898 | EU556032 | ----- | ----- | ----- |
| FEB90 |  | EF537740 | EF537823 | EU556093 | EU555899 | EU556033 | ----- | ----- | ----- |
| FR139 |  | EU555631 | EU555783 | EU556097 | EU555901,EU624451 | EU556037 | ----- | ----- | ----- |
| FR26 |  | EU555636,EU624482 | EU555787 | EU556098 | EU555905,EU624454 | EU556038 | ----- | ----- | ----- |
| JP1 |  | EF537756 | EU555789 | EU556101 | EU555908 | EU556040 | ----- | ----- | ----- |
| LV10 |  | EU555638 | EU555791 | EU556102 | EU555910 | EU556041 | ----- | ----- | ----- |
| LV2 |  | EU555647 | EU555793 | EU556103 | EU555912,EU624456 | EU556042 | ----- | ----- | ----- |
| LV8 |  | EU555648 | EU555795 | EU556104 | EU555914,EU624458 | EU556043 | ----- | ----- | ----- |
| MG2653 | UWBM 60090 | AY881944 | EF537820 | EU556105 | EU555915,EU624459 | EU556044 | ----- | ----- | ----- |
| NE9185 |  | AY881960,AY881961 | EU555799 | EU556107 | EU555917 | EU556047 | ----- | ----- | ----- |
| RU8(WP-137) |  | AY881956,AY881957 | EF537826 | EU556094 | EU555900 | EU556034 | ----- | ----- | ----- |
| RUZM | UWBM 75904 | AY881965,AY881966 | EU555800 | EU556118 | EU555941 | EU556058 | ----- | ----- | ----- |
| SP7385 |  | EU555662 | EU555810 | EU556111 | EU555924 | EU556051 | ----- | ----- | ----- |
| SP1687 |  | EU555654 | EU555805 | EU556109 | EU555926,EU624462 | EU556049 | ----- | ----- | ----- |
| SP1853 |  | EU555659 | EU555808 | EU556110 | EU555932,EU624465 | EU556050 | ----- | ----- | ----- |
| SR11 |  | EF537749 | EF537816 | EU556112 | EU555933 | EU556052 | ----- | ----- | ----- |
| SR16 |  | EF537751 | EF537817 | EU556113 | EU555934 | EU556053 | ----- | ----- | ----- |
| SR3 |  | EF537746 | EF537815 | EU556114 | EU555937 | EU556054 | ----- | ----- | ----- |
| TB1548 | UAM 18111 | EU555664 | EU555812 | EU556115 | EU555938 | EU556055 | ----- | ----- | ----- |
| VO3166 | UWBM 56499 | AY881945 | EU555813 | EU556116 | EU555939 | EU556056 | ----- | ----- | ----- |
| VO5528 | UWBM 56676 | AY881951,AY881952 | EU555814 | EU556117 | EU555940 | EU556057 | ----- | ----- | ----- |
| *Chauna toquata* |  | ----- | ----- | ----- | DQ881793^2^ | ----- | EU739387^3^ | AY274082^4^ | EF521458^5^ |
| *Anseranas semipalmata* |  | ----- | ----- | ----- | ----- | ----- | EU739366^3^ | AY274083^4^ | EF521439^5^ |
| *Dendrocygna bicolor* |  | ----- | ----- | ----- | ----- | ----- | AY695133^6^ | AY274084^4^ |  |
| *Oxyura jamaicensis* |  | ----- | AY747807^7^ | AY747819^7^ | ----- | ----- | EU739453^3^ | AY747848^7^ | EF521520^5^ |
| *Biziura lobata* |  | ----- | ----- | ----- | ----- | ----- | EU739373^3^ | ----- | EF521446^5^ |
| *Malacorhynchus membranaceus* |  | ----- | ----- | ----- | ----- | ----- | EU739433^3^ | ----- | EF521501^5^ |

^1^Peters et al.; AY###### [1], EF###### [2], and EU###### [3]. Cells with two accession numbers indicate that the two alleles were resolved experimentally and each allele was archived separately; ^2^Ericson et al. [4]; ^3^Hackett et al. [5]; ^4^Sorenson et al. [6]; ^5^Yuri et al. [7]; ^6^Fain and Houde [8]; ^7^McCracken and Sorenson [9].

References

1. Peters JL, McCracken KG, Zhuravlev YN, Lu Y, Wilson RE, et al (2005) Phylogenetics of wigeons and allies (Anatidae: *Anas*): The importance of sampling multiple loci and multiple individuals. Mol Phylogenet Evol 35: 209-224.

2. Peters JL, Zhuravlev Y, Fefelov I, Logie A, Omland KE (2007) Nuclear loci and coalescent methods support ancient hybridization as cause of mitochondrial paraphyly between gadwall and falcated duck (*Anas* spp.). Evolution 61: 1992-2006.

3. Peters JL, Zhuravlev YN, Fefelov I, Humphries EM, Omland KE (2008) Multilocus phylogeography of a Holarctic duck: Colonization of North America from Eurasia by gadwall (*Anas strepera*). Evolution 62: 1469-1483.

4. Ericson PGP, Anderson CL, Britton T, Elzanowski A, Johansson US, et al (2006) Diversification of Neoaves: integration of molecular sequence data and fossils. Biol Lett 2: 543-547.

5. Hackett SJ, Kimball RT, Reddy S, Bowie RCK, Braun EL, et al (2008) A phylogenomic study of birds reveals their evolutionary history. Science 320: 1763-1768.

6. Sorenson MD, Oneal E, García-Moreno J, Mindell DP (2003) More taxa, more characters: The hoatzin problem is still unresolved. Mol Biol Evol 20: 1484-1498.

7. Yuri T, Kimball RT, Braun EL, Braun MJ (2008) Duplication and accelerated evolution of growth hormone gene in passerine birds. Mol Biol Evol 25: 352-361.

8. Fain MG, Houde P (2004) Parallel radiations in the primary clades of birds. Evolution 58: 2558-2573. 163.

9. McCracken KG, Sorenson MD (2005) Is homoplasy or lineage sorting the source of incongruent mtDNA and nuclear gene trees in the stiff-tailed ducks (*Nomonyx*-*Oxyura*)? Syst Biol 54: 35-55.
