# Supplementary material for: Heterogeneity in Genetic Diversity among Non-Coding Loci Fails to Fit Neutral Coalescent Models of Population History
Source: PLoS One. 2012 Feb 22;7(2):e31972. doi: 10.1371/journal.pone.0031972 (PMC3285185; doi:10.1371/journal.pone.0031972)
Supplement: Table S3 — Equations for converting parameters estimated in im and lamarc to the appropriate scale for simulating genetic diversity in the program ms. (DOCX) [file pone.0031972.s004.docx]

Table S3. Equations for converting parameters estimated in im and lamarc to the appropriate scale for simulating genetic diversity in the program ms [1]. (See text for additional explanations of the terms in the equations.)

|  | **IM or LAMARC** | **MS** | **Conversion** |
| --- | --- | --- | --- |
| **Effective population size (N_e_)** | θ_IM_ = 4*N_e_u*  Θ_LAMARC_ = 4*N_e_μ* | θ_MS_ = 4*N_e_u* | θ_MS_ = θ_IM_  θ_MS_ = Θ_LAMARC_ × *l* |
| **Migration rates (M)** | *m*_IM_ = *m*/*u*  *M*_lamarc_ = *m*/*μ* | *M*_MS_ = 4*N_e_m* | *M*_MS_ = θ_im_*m*_IM_  *M*_LAMARC_ = Θ_LAMARC_*M*_LAMARC_ |
| **Time since divergence (T)** | *t_I_*_M_=*Tu* | *T*_MS_ = *t*/4*N_e_* | T_MS_ = *t*_IM_/θ_IM_ |
| **Population growth rates (α)** | ---- | α = -ln(θ_t_/θ_0_)/*T*_MS_ | α = -ln(θ_A-IM_/θ_IM_)/*T*_MS_ |
| **Recombination rates (*r*)** | *r*_LAMARC_ = *C*/*µ* | *ρ* = 4*N_e_r*_MS_ | *ρ* = θ_MS_*r*_LAMARC_ |

References

1. Hudson RR (2002) Generating samples under a Wright-Fisher neutral model of genetic variation. Bioinformatics 18: 337-338.
